# *Lactobacillus paracasei* metabolism of rice bran reveals metabolome associated with *Salmonella* Typhimurium growth reduction

**DOI:** 10.1111/jam.13459

**Published:** 2017-05-11

**Authors:** N.J. Nealon, C.R. Worcester, E.P. Ryan

**Affiliations:** ^1^ Department of Environmental and Radiological Health Sciences College of Veterinary Medicine and Biomedical Sciences Colorado State University Fort Collins CO USA

**Keywords:** *L. paracasei* subsp*. paracasei*, metabolomics, prebiotic, probiotic, rice bran, *S*. Typhimurium, synbiotic

## Abstract

**Aims:**

This study aimed to determine the effect of a cell‐free supernatant of *Lactobacillus paracasei* ATCC 27092 with and without rice bran extract (RBE) on *Salmonella* Typhimurium 14028s growth, and to identify a metabolite profile with antimicrobial functions.

**Methods and Results:**

Supernatant was collected from overnight cultures of *L. paracasei* incubated in the presence (LP+RBE) or absence (LP) of RBE and applied to *S*. Typhimurium. LP+RBE reduced 13·1% more *S*. Typhimurium growth than LP after 16 h (*P* < 0·05). Metabolite profiles of LP and LP+RBE were examined using nontargeted global metabolomics consisting of ultra‐high‐performance liquid chromatography coupled with tandem mass spectrometry. A comparison of LP and LP+RBE revealed 84 statistically significant metabolites (*P* < 0·05), where 20 were classified with antimicrobial functions.

**Conclusions:**

LP+RBE reduced *S*. Typhimurium growth to a greater extent than LP, and the metabolite profile distinctions suggested that RBE favourably modulates the metabolism of *L. paracasei*. These findings warrant continued investigation of probiotic and RBE antimicrobial activities across microenvironments and matrices where *S*. Typhimurium exposure is problematic.

**Significance and Impact of the Study:**

This study showed a novel metabolite profile of probiotic *L. paracasei* and prebiotic rice bran that increased antimicrobial activity against *S*. Typhimurium.

## Introduction

Globally, *Salmonella enterica* serovar Typhimurium accounts for the majority of human Salmonellosis cases each year. *Salmonella* Typhimurium causes widespread disease because of zoonotic transmission from cattle, poultry, swine, sheep, rodents and horses (Tsolis *et al*. 1999). An emerging strategy against *S*. Typhimurium involves the use of probiotics and prebiotics. As defined by the World Health Organization and Food and Drug Administration, probiotics are live micro‐organisms that, when administered in adequate amounts, confer a health benefit to the host (Anon 2006). Many widely investigated probiotic species belong to the bacterial genus *Lactobacillus*, which has a natural ability to reduce the growth of *S*. Typhimurium (Heredia‐Castro *et al*. 2015). In particular, *Lactobacillus paracasei* strains have been shown to effectively reduce the growth of enteric pathogens including *S*. Typhimurium, *Listeria monocytogenes* and *Escherichia coli* O157:H7 (Caridi 2002; Chiang and Pan 2012; Valerio *et al*. 2013).

Rice bran, the outer layer of the rice grain, is a natural and rich source of prebiotics that can be metabolized by the gut microbiome to modulate mucosal immune responses, reduce intestinal colonization of enteric pathogens (Kumar *et al*. 2012) and increase numbers of native probiotic lactobacilli (Henderson *et al*. 2012; Goodyear *et al*. 2015). Rice bran was also shown to support probiotic *E. coli* Nissle and *Lactobacillus rhamnosus* GG growth in gnotobiotic piglets (Yang *et al*. 2015).

Molecular mechanisms by which *Lactobacillus* sp. utilize and metabolize prebiotics are not well understood. Metabolomics has been minimally used to elucidate the functional significance of synbiotics via identification and quantification of small molecules (Ryan *et al*. 2011). Past investigations evaluated small‐molecule profiles produced by the microbiome *in vitro* and *in vivo*, including probiotics (Vitali *et al*. 2010; Mozzi *et al*. 2013; Weir *et al*. 2013). Metabolite profiles have been determined for an increasing number of foods fermented by lactic acid bacteria including rye, yogurt and wine (Settachaimongkon *et al*. 2014; Arbulu *et al*. 2015; Koistinen *et al*. 2016.).

It was hypothesized that a combination of *L. paracasei* and rice bran extract (RBE) would result in a unique profile of metabolites with antimicrobial activity that more effectively reduce the growth of *S*. Typhimurium compared to *L. paracasei* alone. This study aimed to compare the effectiveness of *L. paracasei* alone and *L. paracasei* with RBE supernatants at reducing *S*. Typhimurium growth and to evaluate their metabolomic profiles. The small molecule changes that occur in the presence and absence of RBE illustrate how *L. paracasei* and rice bran synergistically promote *S*. Typhimurium growth reduction.

## Materials and methods

### Bacterial strains and culture reagents

*Lactobacillus paracasei* ATCC 27092 was purchased from ATCC (Manassas, VA), and *S. enterica* subsp. *enterica* serovar Typhimurium 14028s Kan^r^ (rPSM::GFP) was a generous gift from Dr Andres Vazquez‐Torres (University of Colorado). All bacterial cultures were stored at −80°C as 1‐ml aliquots supplemented with 20% glycerol in Luria–Bertani (LB) broth (MO BIO Laboratories, Inc. Carlsbad, CA) for *S*. Typhimurium, and deMan Rogosa Sharpe broth (MRS) (Becton, Dickinson, and Company Difco Laboratories, Franklin Lakes, NJ) for *L. paracasei*. MRS broth and agar, LB broth and MacConkey agar (Becton, Dickinson, and Company Difco Laboratories) were prepared and sterilized according to the manufacturer. To prepare MRS broth supplemented with RBE, 100 *μ*g ml^−1^ RBE was added to the broth. The media was autoclaved with an 18‐min sterilization time, then stored at 4°C until use.

### Rice bran extraction

RBE was prepared as described previously (Kumar *et al*. 2012). Briefly, 4 g of finely ground, heat‐stabilized Calrose rice bran (USDA‐ARS Rice Research Unit, Stuttgart, AK) was extracted in 42·6 ml of 80% methanol. The mixture was vortexed (232 Vortexer Fisher Scientific, Pittsburgh, PA, USA) on a high power setting for 5 min, incubated at −80°C overnight and centrifuged (Beckman Coulter Allegra X‐14R, Indianapolis, IN, USA) at 3724 ***g*** for 5 min. The supernatant was collected, and kept at −80°C until it could be dried in a speedvac concentrator (SPD1010; Thermo Scientific, Pittsburgh, PA, USA) at 45°C, with the heating time for 5 min, and a vacuum pressure of 7·5 torrs.

### *L. paracasei* cell‐free supernatant preparation

The cell‐free supernatant (CFS) preparation was modified from a published procedure (Wang *et al*. 2012). Briefly, *L. paracasei* isolates were thawed from storage in −80°C, suspended in MRS broth and grown at 37°C until mid/late logarithmic phase. Approximately 1 × 10^7^ cells were inoculated into 15 ml of MRS or MRS + 100 *μ*g ml^−1^ RBE. The RBE concentration was determined based on dose–response experiments that observed 100 *μ*g ml^−1^ of RBE, when added to fixed amounts of *S*. Typhimurium, reduced growth compared to a RBE‐free control culture of *S*. Typhimurium, as evidenced by differences in optical density at 600 nm (OD600) over 12 h (Fig.**** S1a). Each treatment was incubated at 37°C for 24 h. *Lactobacillus paracasei* supernatant (LP) and *L. paracasei* supernatant with RBE (LP+RBE) was collected by centrifuging two times at 3724 ***g*** for 10 min. The pH of the supernatant was adjusted using a pH meter (Corning Pinnacle 530, Cole‐Parmer, Vernon Hills, IL, USA) with 1 mol l^−1^ NaOH (Sigma Aldrich) until a pH of 4·5 was reached. CFS was filter‐sterilized through a 0·2‐*μ*m pore (Pall Corporation LifeSciences Acrodisc syringe filters, Port Washington, NY, USA) into 1‐ml aliquots before being stored at −80°C. Sterility of all CFS was confirmed by plating the treatment on MRS agar and confirming the absence of any bacterial growth after 48 h. Three independent batches of CFS were used in this study.

### *S. Typhimurium* growth reduction assay

*Salmonella* Typhimurium was thawed from storage in −80°C and suspended in sterile LB. Stocks were grown in a 24‐well plate at 37°C until early/mid logarithmic phase, and were assessed using the Cytation3 plate reader (BioTek Instruments Inc., Winooski, VT, USA). In a 96‐well plate, 20 *μ*l of *S*. Typhimurium (approx. 2 × 10^6^ cells) was diluted 10‐fold into 180 *μ*l of sterile LB. To determine an appropriate treatment volume for the growth reduction assay, a dose curve analysis using LP was performed on *S*. Typhimurium (Fig. S1b). In brief, 100, 50 and 25 *μ*l doses of 100% LP were added to approx. 2 × 10^5^ *S*. Typhimurium cells, and growth was evaluated for 24 h at OD600. Fifty microlitres of treatment was the lowest dose at which a difference was observed in the inhibitory capacity of LP compared to a volume‐adjusted control.

To evaluate the effect of CFS on *S*. Typhimurium growth, a 50 *μ*l of treatment with different percentages of CFS were added to each well: 0% vehicle control, 20, 40, 60, 80 and 100%. A negative control of *S*. Typhimurium inoculated with 50 *μ*l LB broth was included on each well plate. To control for pH differences between treatments, all media was adjusted to a pH of 4·5 using 1N of NaOH or HCl before use. The plate was read on an automatic plate reader, incubated at 37°C for approx. 16 h, and individual wells were read at OD600 every 20 min. To evaluate differences between all treatments, OD600 was plotted over time. To further quantify growth reduction by LP and LP+RBE, per cent growth reduction was calculated from OD600 values using the following equation:

$$\frac{100\%\;\text{supernatant}-\text{vehicle control}}{\text{vehicle control}}\times 100\%$$

Experiments were repeated four times for all CFS concentrations, eight times for 100% LP and nine times for 100% LP+RBE using the same three batches of supernatant as described previously.

### Probiotic supernatant agar well diffusion assay

The agar well diffusion assay was modified from a published procedure (Aminnezhad *et al*. 2015). Briefly, Mueller–Hinton agar (Hardy Diagnostics, Santa Maria, CA) was inoculated with *S*. Typhimurium suspended in normal saline (0·89% g NaCl ml^−1^), equivalent to a 0·5 McFarland Standard (Hardy Diagnostics). Wells (8·0 mm) were punched into the agar and filled with 100 *μ*l of the following treatments adjusted to a final pH of 4·5: vehicle control, vehicle control+RBE, LP and LP+RBE. Non‐pH‐adjusted normal saline was included as a negative control on each plate. Plates were incubated at 37°C overnight, and inhibition zone diameters were measured in millimetres. Measurements were collected from seven different plates and included supernatant collected from three independent cultures of LP and LP+RBE.

### Supernatant extract preparation for metabolomics

Metabolomics was performed by Metabolon Inc © (Durham, NC). Briefly, 1 ml of supernatant samples were stored at −80°C in microcentrifuge tubes, and sent on dry ice to Metabolon in triplicate. The samples sent for analysis included vehicle control, vehicle control+RBE, LP and LP+RBE. Upon arrival, samples were stored at −80°C in liquid nitrogen until processing. To improve recovery of small molecules prior to detection, the protein fraction was removed by extracting the sample with a 5 : 1 methanol : water solution, using vigorous shaking at room temperature for 2 min followed by centrifugation at 680 ***g*** for 3 min. The extracted samples were split into four parts for analysis via ultra‐high‐performance liquid chromatography–tandem mass spectrometry (UPLC‐MS/MS) including two separate reverse phase UPLC‐MS/MS with positive ion mode electrospray ionization (ESI), reverse phase UPLC‐MS/MS with negative ion mode ESI and one sample for high liquid chromatography UPLC‐MS/MS‐negative ion mode ESI.

### UPLC‐MS/MS analysis

Metabolite profiling was performed using a Waters ACQUITY UPLC, a Thermo Scientific (Waltham, MA, USA) Q‐Exactive heated electrospray ionization (HESI‐II) source, and an Orbitrap mass analyser operated at 35 000 mass resolution. For UPLC analysis, the sample extracts were dried and reconstituted in solvents appropriate for each of the four detection methods, and standards were included to ensure experimental consistency. Acidic positive ion conditions were optimized for either hydrophobic or hydrophilic compounds and were eluted from a C18 column (Waters UPLC BEH C18‐2·1 × 100 mm, 1·7 μm) using water and methanol (hydrophilic optimization) or methanol, acetonitrile and water (hydrophobic optimization) containing 0·05% perfluoropentanoic acid (PFPA) (hydrophilic optimization) or 0·5% PFPA (hydrophobic optimization) and 0·1% formic acid. Two aliquots were analysed using basic negative ion conditions; one was eluted on a separate C18 column using methanol and water with 6·5 mmol l^−1^ of ammonium bicarbonate at pH 8, and the other was eluted from a HILC column (Waters UPLC BEH Amide 2·1 × 150 mm, 1·7 *μ*m) using a water–acetonitrile gradient with 10 mmol l^−1^ of ammonium formate, pH 10·8. The total scan range covered 70–1000 *m*/*z*.

### Data extraction and compound identification

Raw data were extracted, peak‐identified and quality‐control processed as previously described (Brown *et al*. 2016) and compounds were then identified by comparison to library entities of purified standards or recurrent unknown entities, including over 3300 commercially available purified standard compounds. Identifications were made based on retention time/index with a narrow window of identification, mass to charge ratio (*m*/*z*) ±10 parts per million and chromatographic data including MS/MS forward and reverse scores between the experimental data and authentic standards. The raw counts of both supernatant profiles were converted into relative abundances and then median‐scaled to one. For each metabolite, fold difference (FD) was calculated by dividing the scaled relative abundance of LP+RBE by LP.

### Metabolic pathway analysis

Pathway analysis was conducted as described previously (Brown *et al*. 2016). For selected lipids and amino acids/peptides, metabolite FD between LP+RBE and LP were visualized using Cytoscape 2.8.3 software. For each metabolite, node colour was determined by the direction of FD when comparing LP+RBE to LP, and node diameter was determined by the magnitude of the FD. The value on each node represents a pathway enrichment score, which was calculated by dividing the number of significant metabolites in pathway (*k*) by the total number of detected metabolites in pathway (*m*). This value was then divided by the fraction of the total number of significant metabolites in the data set (*n*) over the total number of detected metabolites in the complete data set (*N*):

$$\frac{k/m}{n/N}$$

Pathway enrichment scores greater than one indicated that a given pathway contained more metabolites with statistically significant FD between LP+RBE and LP compared to all pathways in the study.

### Statistical analysis

All statistical analyses for the *S*. Typhimurium growth reduction and well diffusion assays were performed using GraphPad Prism 6.07 (San Diego, CA). For the *S*. Typhimurium growth reduction assays, treatments were analysed using a two‐way repeated measures anova with a Bonferroni post‐test to compare treatment means. For the agar well diffusion assay, treatments were compared using a one‐way anova with a Tukey post‐test to compare treatment means. Significance was determined at the level of *P* < 0·05. For metabolomic data, statistical analysis was performed by Metabolon Inc using ArrayStudio (Omnicsoft, Cary, NC) R ver. 2.142 and/pr SAS ver. 9.4. The relative abundance of each metabolite from LP and LP+RBE was scaled to that metabolite's median relative abundance and each scaled relative abundance was compared between LP and LP+RBE using a Welch's two‐sample *t* test. Statistical significance was determined at the level of *P* < 0·05.

## Results

### *S. Typhimurium* growth reduction by *L. paracasei* and rice bran extract

**Figure 1:**
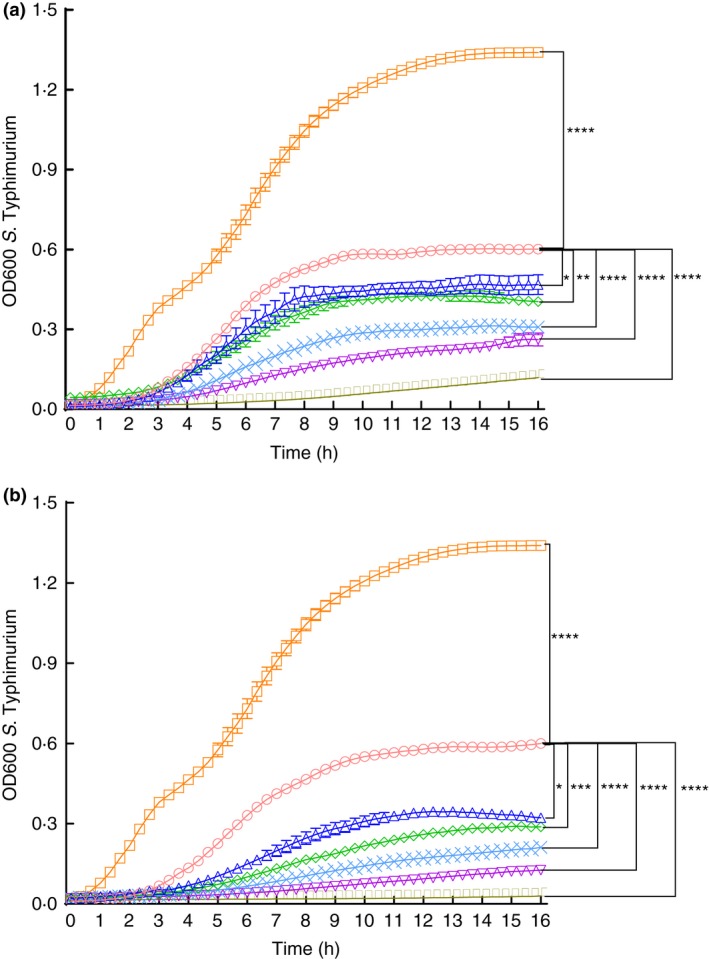
*Lactobacillus paracasei* supernatant in the presence and absence of rice bran extract reduces *S*. Typhimurium growth in a dose‐dependent manner. The dose‐dependent effects of (a) LP and (b) LP+RBE on *S*. Typhimurium growth were evaluated. Optical density at a wavelength of 600 nm was plotted on the *y*‐axis and time on the *x*‐axis. Each point represents the mean of four independent experiments and error bars indicate standard error. □ indicates negative control, ○ indicates vehicle control or vehicle control+RBE, ▵ indicates 20% LP or LP+RBE, ◊ indicates 40% LP or LP+RBE, X indicates 60% LP or LP+RBE, ▽ indicates 80% LP or LP+RBE, and ▯ indicates 100% LP or LP+RBE. Statistical significance between all treatments compared to the vehicle control occurred at (a) 5·0 h in LP and (b) 3·7 h in LP+RBE where *P < 0·05, **P < 0·01, ***P < 0·001 and ****P < 0·0001. In (a) and (b), results represent four independent experiments. [Colour figure can be viewed at wileyonlinelibrary.com]

The dose‐dependent effects of LP and LP+RBE on *S*. Typhimurium growth were determined with increasing percentages of supernatant that were added and compared to a fixed volume of a vehicle control or vehicle control+RBE respectively. In both the LP and LP+RBE treatments, *S*. Typhimurium growth was reduced in a dose‐dependent manner (Fig. 1). By 5·0 h, all LP doses (20–100%) significantly reduced *S*. Typhimurium growth compared to the vehicle control. The average percent difference between LP and the vehicle control included: 22·2% for the 20% LP dose, 35·9% for 40% LP, 47·4% for 60% LP, 55·3% for 80% LP and 60·6% for the 100% LP (Fig. 1a). By the study endpoint of 16 h, two LP concentrations continued to show increased growth reduction compared to the vehicle control and included: 40% LP (41·7%) and 100% LP (68·4%) (Fig. 1a)**.**

LP+RBE treatments significantly reduced *S*. Typhimurium growth by 3·7 h. The average per cent difference for LP+RBE over RBE alone was 8·9% for the 20% LP+RBE dose, 16·1% for 40% LP+RBE, 21·6% for 60% LP+RBE, 25·5% for 80% LP+RBE and 37·0% for 100% LP+RBE (Fig. 1b). At 16 h, all LP+RBE doses showed increasing growth reduction: 38·8% for 20% LP+RBE, 43·9% for 40% LP+RBE, 55·2% for 60% LP+RBE, 67·6% for 80% LP+RBE and 82·0% for 100% LP+RBE (Fig. 1b). At 16 h, the 100% CFS dose showed highest *S*. Typhimurium growth reduction compared to the vehicle control: 68·4% for 100% LP and 82·0% for 100% LP+RBE (Fig. 1)

**Figure 2:**
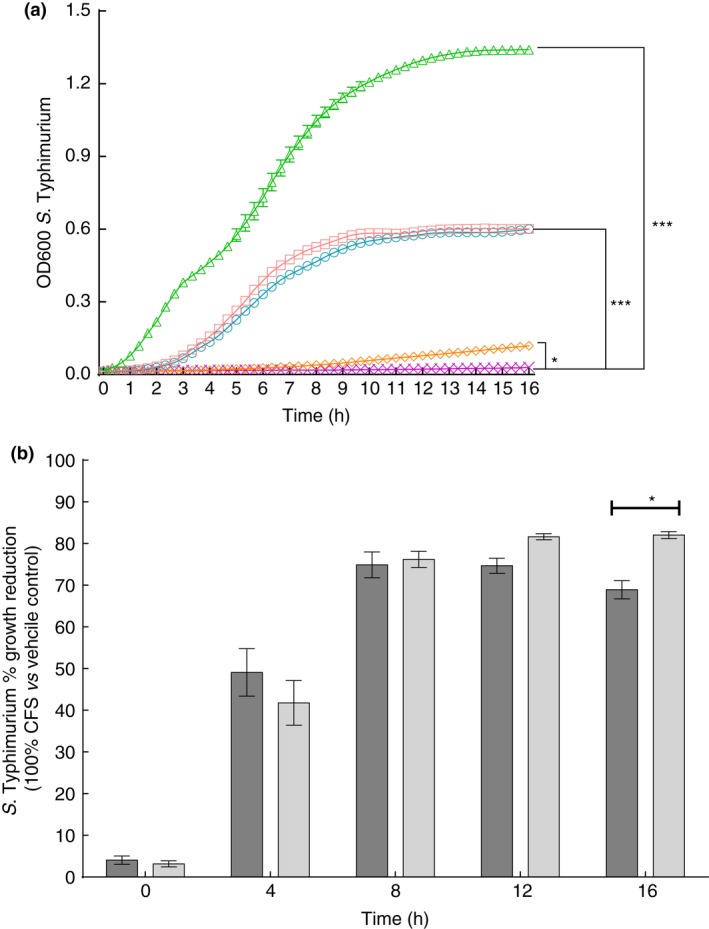
Rice bran extract enhances the ability of *Lactobacillus paracasei* supernatant to reduce *S*. Typhimurium growth. (a) The effectiveness of 100% LP and 100% LP+RBE at reducing *S*. Typhimurium growth were compared. The optical density at 600 nm is plotted on the *y*‐axis, and time on the *x*‐axis. Each point represents the mean of eight (LP) or nine independent experiments (LP+RBE) treatments and error bars indicate SE ▵ indicates negative control, □ indicates vehicle control, ○ indicates vehicle control+RBE, ◊ indicates LP and X indicates LP+RBE. By 9·0 h, LP+RBE reduced *S*. Typhimurium growth more than LP, vehicle control, vehicle control+RBE and the negative control, where*P < 0·05, ***P < 0·001 and ****P < 0·0001. (b) For the 100% CFS treatments, per cent *S*. Typhimurium growth inhibition of LP and LP+RBE treatments media was compared at 0, 4·0, 8·0, 12·0 and 16·0 h. At 16·0 h, LP+RBE reduced growth 13·1% more than LP *P < 0·05. Each bar represents the mean per cent difference of eight (LP) or nine (LP+RBE) independent experiments and error bars indicate standard error. Light grey bars represent LP and dark grey bars represent LP+RBE. [Colour figure can be viewed at wileyonlinelibrary.com]

Next, 100% LP and 100% LP+RBE were compared to each other. At 9·0 h, LP+RBE was more effective than LP at reducing *S*. Typhimurium growth (*P* < 0·05) and became increasingly effective through 16 h (*P* < 0·0001) (Fig. 2a). The 100% LP+RBE reduced *S*. Typhimurium growth 13·1% more than 100% LP at 16 h (*P* < 0·05) (Fig. 2b). All LP+RBE doses (20, 40, 60, 80%) were more effective than LP at reducing *S*. Typhimurium growth by 16 h (data not shown).

### Probiotic supernatant agar well diffusion assay against *S*. Typhimurium

To further assess the ability of CFS to reduce *S*. Typhimurium growth, the inhibitory zone diameters of LP, LP+RBE, vehicle control, vehicle control+RBE and normal saline (negative control) treatments were compared in an agar well diffusion assay where all treatments were pH adjusted to 4·5 prior to use. The mean inhibitory zone diameter (millimetres) and standard error are displayed for each treatment in Table 1 and represent seven independent experiments: normal saline (8·00 ± 0·00), vehicle control (8·93 ± 0·468), vehicle control+RBE (9·43 ± 0·517), LP (10·86 ± 0·261) and LP+RBE (12·07 ± 0·277). The diameters of the wells themselves were 8·0 mm, and normal saline did not create an inhibitory zone in any replicate. LP+RBE had a larger inhibitory zone diameter than all treatments: LP+RBE *vs* normal saline, vehicle control and vehicle control+RBE (*P* < 0·001), LP+RBE *vs* LP (*P* < 0·05).

**Table 1:** Agar well diffusion of *Lactobacillus paracasei* supernatant against *Salmonella* Typhimurium in the presence and absence of rice bran extract

Treatment	Zone of Inhibition (mm)^a^
Normal saline (negative control)	8·00 ± 0·00
Vehicle control	8·93 ± 0·468
Vehicle control+RBE	9·43 ± 0·517
LP	10·86 ± 0·261
LP+RBE	12·07 ± 0·277

a Inhibitory zone diameters (millimetres) against *S*. Typhimurium were determined for supernatants and control media. Results are reported as (mean ± standard error) and were collected from seven independently measured plates. Measurements were analysed using a one‐way anova, and pairs of treatments were compared using a Tukey post‐test, where statistical significance was determined as *P* < 0·05. LP+RBE had a significantly larger zone of inhibition compared to all other treatments: Normal saline (*P* < 0·0001), vehicle control (*P* < 0·0001), vehicle control+ RBE (*P* < 0·0001), LP (*P* < 0·05).

### Metabolomics of probiotic *L. paracasei* supernatant and rice bran extract

**Figure 3:**
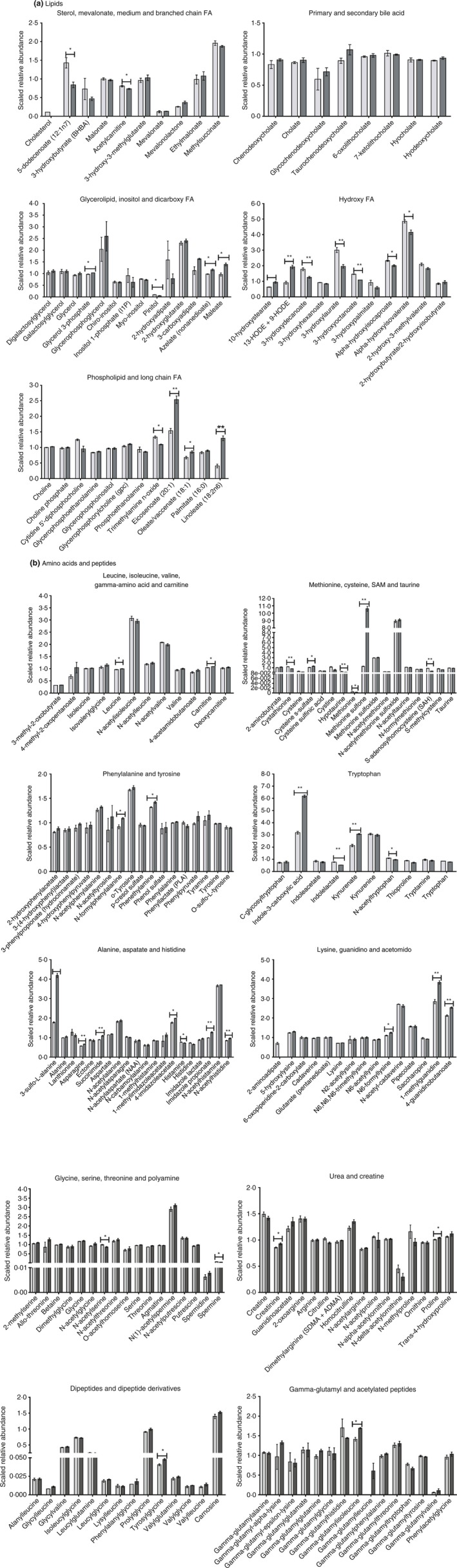
Rice bran extract alters the lipid, amino acid and peptide metabolite profiles of *Lactobacillus paracasei* supernatant. LP and LP+RBE were profiled using UPLC‐MS/MS. Each bar represents three independent samples and depicts the metabolite scaled relative abundance. Error bars depict standard error and *P < 0·05, and **P < 0·01. Metabolites were classified into metabolic pathways of (a) lipids and (b) amino acids/peptides based on their biochemical properties and/or physiological functions. Light grey bars represent LP and dark grey bars represent LP+RBE. FA indicates fatty acid and SAM indicates S‐adenosyl methione.

Metabolome analysis of LP and LP+RBE led to identification of 362 metabolites that were organized by chemical class and summarized in Table**** 2. Of the 362 metabolites, 138 were classified as amino acids, 29 peptides, 29 carbohydrates, 11 TCA cycle, 54 lipids, 54 nucleotides, 20 cofactors and vitamins and 27 phytochemicals. There were a total of 84 metabolites with a FD that significantly differed between LP+RBE and LP (Table 3). Of these, 58 metabolites that had a higher and 26 had a lower relative abundance in LP+RBE compared to LP. Amino acid and lipid metabolite classes represented ~55% of metabolites differentially expressed between LP and LP+RBE. The relative abundance distributions for lipids and amino acids/small peptides across groups are depicted in Fig. 3.

**Table 2:** Number of metabolites across classes that show a higher or lower fold change in *Lactobacillus paracasei* supernatant cultured in the presence or absence of rice bran extract

Metabolite classification	Number of metabolites (*P* < 0·05)
Amino acids	138 (19↑, 9↓)
Peptides	29 (2↑)
Carbohydrates	29 (7↑)
TCA cycle	11 (2↑, 1↓)
Lipids	54 (8↑, 9↓)
Nucleotides	54 (12↑, 3↓)
Cofactor and vitamins	20 (1↑, 1↓)
Other phytochemicals	27 (7↑, 3↓)
Total number of identified metabolites	362 (58↑, 26↓)

Metabolite profiles of *L. paracasei* (LP) and *L. paracasei* + rice bran extract (LP+RBE) supernatant. For each metabolite, fold difference was calculated by dividing the scaled relative abundance in LP+RBE by LP. Fold differences were analysed using a Welch's two‐sample *t*‐test, and metabolites with a fold difference at statistically different (*P* < 0·05) levels between LP+RBE and LP are marked with ↑ or ↓ to denote the direction of change when comparing LP+RBE to LP.

**Table 3:** Statistically significant metabolites from *Lactobacillus paracasei* supernatant prepared in the presence and absence of rice bran extract

Metabolite	HMDB*	Fold difference^a^	*P*‐value^b^
*Amino acid*			
Methionine sulfone	–	↑8·29	3·16 × 10^−6^
3‐sulfo‐L‐alanine	02757	↑2·34	3·13 × 10^−5^
Indole‐3‐carboxylic acid	03320	↑1·94	0·00065
Kynurenate	00715	↑1·44	0·0086
1‐methylguanidine	01522	↑1·34	0·0063
Imidazole propionate	02271	↑1·28	0·0047
Cysteine s‐sulphate	00731	↑1·24	0·013
Succinimide	–	↑1·22	0·0075
Beta‐alanine	00056	↑1·22	0·0089
4‐guanidinobutanoate	03464	↑1·19	0·0024
*N*‐formylphenylalanine	–	↑1·18	0·048
Asparagine	00168	↑1·17	0·0015
*N*‐acetylhistidine	32055	↑1·13	0·020
N6‐formyllysine	–	↑1·13	0·013
4‐imidazoleacetate	02024	↑1·12	0·024
Phenethylamine	02017	↑1·07	0·030
Creatinine	00562	↑1·08	0·037
Carnitine	00062	↑1·03	0·034
Proline	00162	↑1·03	0·040
Leucine	00687	↑1·03	0·042
Histamine	00870	↓0·94	0·042
Acetylcarnitine	00201	↓0·90	0·042
*N*‐acetyltryptophan	13713	↓0·88	0·030
*N*‐acetylserine	02931	↓0·87	0·037
Spermine	01256	↓0·70	0·044
Indolelactate	00671	↓0·68	0·0040
Cystathionine	00099	↓0·67	0·00019
Methionine	00696	↓0·64	0·023
Hypotaurine	00965	↓0·46	0·0037
S‐adenosylhomocysteine (SAH)	00939	↓0·39	0·0010
*Peptide*			
cGamma‐glutamylisoleucine	11170	↑1·19	0·024
Tyrosylglycine	–	↑1·17	0·017
*Carbohydrate*			
Sucrose	00258	↑7·39	5·76 × 10^−7^
Arabonate/xylonate	–	↑1·87	0·0059
Ribonate	00867	↑1·86	0·0015
Erythrulose	06293	↑1·46	0·029
Erythronate	00613	↑1·24	0·016
*N*‐acetylglucosamine/*N*‐acetylgalactosamine	–	↑1·12	0·018
Glycerate	00139	↑1·07	0·0026
*TCA cycle*			
Tricarballylate	31193	↑1·79	6·83 × 10^−5^
Citraconate/glutaconate	–	↑1·16	0·017
Alpha‐ketoglutarate	00208	↓0·76	0·031
*Lipid*			
Linoleate (18:2n6)	00673	↑3·18	0·0075
13‐HODE + 9‐HODE	–	↑2·11	0·0082
Eicosenoate (20:1)	02231	↑1·65	0·0017
10‐hydroxystearate	–	↑1·49	0·014
Maleate	00176	↑1·44	0·017
Oleate/vaccenate (18:1)	–	↑1·26	0·041
Azelate (nonanedioate)	00784	↑1·18	0·024
Glycerol 3‐phosphate	00126	↑1·05	0·031
Alpha‐hydroxyisocaproate	00746	↓0·86	0·016
Alpha‐hydroxyisovalerate	00407	↓0·85	0·025
Trimethylamine N‐oxide	00925	↓0·82	0·014
3‐hydroxyoctanoate	01954	↓0·74	0·0015
3‐hydroxydecanoate	02203	↓0·70	0·0085
3‐hydroxylaurate	00387	↓0·65	0·0055
Pinitol	34219	↓0·61	0·015
5‐dodecenoate (12:1n7)	00529	↓0·58	0·015
*Nucleotide*			
2′‐deoxycytidine	00014	↑1·60	0·0034
N6‐succinyladenosine	00912	↑1·51	0·0032
2′‐deoxyadenosine	00101	↑1·31	0·0015
Orotate	00226	↑1·21	0·023
Guanine	00132	↑1·16	0·0026
Guanosine	00133	↑1·12	0·015
Guanosine‐2′,3′‐cyclic monophosphate	11629	↑1·11	0·046
Hypoxanthine	00157	↑1·08	0·046
Cytidine	00089	↑1·06	0·035
cguanosine 2′‐monophosphate (2′‐GMP)	–	↑1·06	0·036
2′‐deoxyuridine	00012	↑1·05	0·043
Cytidine 3′‐monophosphate (3′‐CMP)	–	↓0·94	0·015
Cytidine 5′‐monophosphate (5′‐CMP)	00095	↓0·91	0·0071
Adenosine	00050	↓0·89	0·0015
*Cofactor and vitamin*			
Pyridoxate	00017	↑1·19	0·020
Pyridoxamine	01431	↓0·80	0·026
*Phytochemicals/Other*			
Beta‐guanidinopropanoate	13222	↑1·74	0·00042
Harmane	–	↑1·62	7·51 × 10^−5^
Nicotianamine	–	↑1·52	0·029
4‐hydroxybenzoate	00500	↑1·40	0·00062
Salicylate	01895	↑1·25	0·019
Pyrraline	–	↑1·24	0·0057
2‐oxindole‐3‐acetate	–	↑1·22	0·019
Daidzein	03312	↓0·86	0·040
2‐ketogluconate	–	↓0·68	0·0066
*N*‐glycolylneuraminate	00833	↓0·61	0·011

*Human metabolome database (HMDB) numbers are given when available.

a For each metabolite, fold difference is expressed as the scaled relative abundance in LP+RBE over LP. Arrows indicate the direction of change between treatments.

b Each metabolite presented has a statistically significant (*P* < 0·05) fold difference, as determined by a Welch's two‐sample *t*‐test.

c Indicates compounds that have not been officially confirmed based on a standard, but second‐order identity in Metabolon Inc. library.

**Figure 4:**
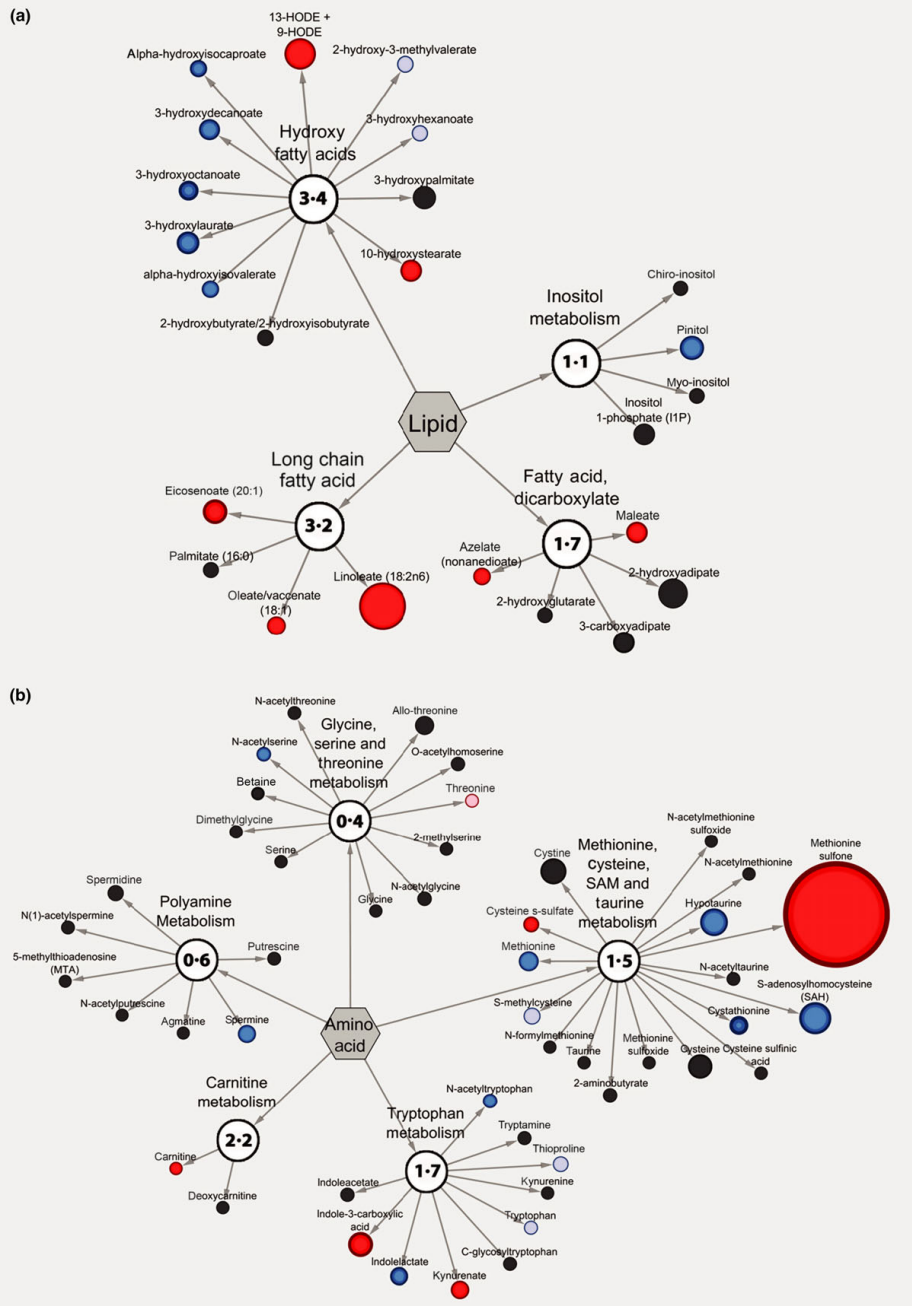
Cytoscape visualization illustrates the metabolic pathways most differentially regulated between treatments. Cytoscape visualization of (a) lipid and (b) amino acid metabolic pathways that differ between LP and LP+RBE. For each metabolite, node diameter is proportional to the fold difference in LP+RBE compared to LP. Node colours indicate the direction of a metabolite's fold difference, where red indicates metabolites with a higher scaled abundance in LP+RBE (*P* < 0·05), blue indicates lower abundance in LP+RBE (*P* < 0·05), pink indicates trending higher in LP+RBE (0·05 < *P* < 0·10), and light blue indicates trending lower in LP+RBE (0·05 < *P* < 0·10). Black nodes indicate metabolites with fold differences that were not significantly altered between treatments. The pathway enrichment score is the number in the circles for each subclassification.

The lipid metabolite changes included, but were not limited to medium‐chain, branched chain, long‐chain and other fatty acids (Fig. 4a). FD between lipids, when comparing LP+RBE to LP included: linoleate (3·18 FD), 13‐HODE + 9‐HODE (2·11 FD), eicosenoate (1·65 FD), 10‐hydroxystearate (1·49 FD), maleate (1·44 FD), oleate/vaccenate (1·26 FD), azelate (1·18 FD), glycerol 3‐phosphate (1·05 FD), alpha‐hydroxyisocaproate (0·86 FD), alpha‐hydroxyisovalerate (0·85 FD), trimethylamine *N*‐oxide (0·82 FD), 3‐hydroxyoctanoate (0·74 FD), 3‐hydroxydecanoate (0·70 FD), 3‐hydroxylaurate (0·65 FD), pinitol (0·61 FD) and 5‐dodecenoate (0·58 FD).

Fold differences between amino acids/peptides when comparing LP+RBE to LP included: methionine sulfone (8·29 FD), 3‐sulfo‐l‐alanine (2·34 FD), indole‐3‐carboxylic acid (1·94 FD), kynurenate (1·44 FD), 1‐methylguanidine (1·34 FD), imidazole propionate (1·28 FD), cysteine s‐sulphate (1·24 FD), succinimide (1·22 FD), 4‐guanidinobutanoate (1·19 FD), *N*‐formylphenylalanine (1·18 FD), asparagine (1·17 FD), *N*‐acetylhistidine (1·13 FD), N6‐formyllysine (1·13 FD), 4‐imidazoleacetate (1·12 FD), phenethylamine (1·07 FD), creatinine (1·08 FD), carnitine (1·03 FD), proline (1·03 FD), leucine (1·03 FD), histamine (0·94 FD), acetylcarnitine (0·90 FD), *N*‐acetyltryptophan (0·88 FD), *N*‐acetylserine (0·87 FD), spermine (0·70 FD), indolelactate (0·68 FD), cystathionine (0·67 FD), methionine (0·64 FD), hypotaurine (0·46 FD) and S‐adenosylhomocysteine (0·39 FD) (Fig. 4b). A literature search of the significant metabolites in the comparison of LP+RBE to LP revealed metabolites with established antimicrobial activities, many of which included the above lipids and amino acids/peptides (Table 4).

**Table 4:** Metabolites from *Lactobacillus paracasei* supernatant prepared in the presence and absence of rice bran extract with reported antimicrobial activities

	Fold difference^a^	*P* value^b^	Functions	References
*Amino acids/peptides*				
Methionine sulfone	↑8·29	3·16 × 10^−6^	Inhibits *S*. Typhimurium growth by blocking glutamate synthesis	Hentchel and Escalante‐Semerena (2015)
Indole‐3‐carboxylic acid	↑1·94	0·00065	Inhibited the growth of *Staphylococcus aureus*,* Pseudomonas aeruginosa*,* Candida albicans*,* Escherichia coli*,* Klebsiella pneumoniae* and multidrug resistant strains of these species	Hinton and Ingram (2000)
Carnitine	↑1·03	0·034	Inhibited the growth of *Streptococcus agalactiae in vitro*.	Atroshi *et al*. (1998)
N‐acetylserine	↓ 0·87	0·037	May increase the susceptibility of *S*. Typhimurium to methicillin *in vitro* by modifying cysteine metabolism	Oppezzo and Anton (1995)
Spermine	↓0·70	0·044	May increase susceptibility of *S*. Typhimurium and other bacteria to Beta‐lactam antibiotics	Kwon and Lu (2007)
S‐adenosylhomocysteine (SAH)	↓0·39	0·001	In high enough concentrations it can inhibit bacterial S‐adenosyl methionine metabolism, which is important for quorum sensing and polyamine synthesis	Simms and Subbaramaiah (1991), Parveen and Cornell (2011)
*TCA cycle*				
Tricarballylate	↑1·79	6·83 × 10^−5^	Toxic to *S. enterica* if accumulated to high levels.	Boyd *et al*. (2012)
*Lipids*				
3‐hydroxyoctanoate	↓0·74	0·0015	It and its derivatives inhibited the growth of *S*. Typhimurium, *E. coli*,* S. aureus*,* L. monocytogenes*,* P. aeruginosa*,* C. albicans* and *Microsporum gypseum*.	Radivojevic *et al*. (2016)
Linoleate (18:2n6)	↑3·18	0·0075	Bactericidal and bacteriostatic against *S. aureus*,* Streptococcus pyogenes* and *E. coli* by inhibiting fatty acid synthesis	Zheng *et al*. (2005)
13‐Hydroxyoctadecadienoate and 9‐Hydroxy‐10,12‐octadecadienoate	↑2·11	0·0082	Detected in an oxylipin extract that inhibited growth of *Penicillium funiculosum* and *Enterococcus hirae in vitro*	Martin‐Arjol *et al*. (2010)
Maleate	↑1·44	0·017	Reduced the growth of *S*. Typhimurium on refrigerated turkey frankfurters	Gadang *et al*. (2008)
coleate/vaccenate (18:1)	↑1·26	0·041	Reduced growth of pathogens on poultry skin, including *S*. Typhimurium and also prevented fatty acid elongation in a variety of pathogens	Zheng *et al*. (2005), Hinton and Ingram (2000)
Azelate (nonanedioate)	↑1·18	0·024	Reduced growth of *S. aureus*,* Staphylococcus epidermidis*, and *Propionibacterium acnes in vitro*	Charnock *et al*. (2004)
Alpha‐hydroxyisocaproate	↓0·86	0·016	Bactericidal against several strains of both Gram‐negative and Gram‐positive bacteria *in vitro*	Sakko *et al*. (2012)
Pinitol	↓0·61	0·015	Synergistically enhances the potency of beta‐lactam antimicrobials by lowering the effective dose eightfold. As part of an *Ademsia aegiceras* extract, it had bacteriostatic and/or bactericidal effects against *S. aureus*,* E. coli*,* Proteus mirabilis* and *Pseudomonas aeruginosa*	Ahmad *et al*. (2016)
*Nucleotides*				
cHypoxanthine	↑1·08	0·046	Inhibits *Salmonella enteriditis* and *Escherichia coli* growth when added to breast milk	Al‐Shehri *et al*. (2015)
*Other phytochemicals*				
Harmane	↑1·62	7·51 × 10^−5^	Inhibits the growth of and functions as a bactericidal agent against *Salmonella* spp. and other pathogens *in vitro* by intercalating with DNA	Cowan (1999), Arshad *et al*. (2008)
4‐hydroxybenzoate	↑1·40	0·00062	Inhibited the growth of *S. aureus*,* E. coli*,* Saccharomyces cerevisiae* and *Fusarium culmorum in vitro*	Kosová *et al*. (2015)
Daidzein	↓0·86	0·040	Extracted from soy milk, where it inhibited the growth of *S*. Typhimurium, and a variety of enteric pathogens	Chin *et al*. (2012)
2‐ketogluconate	↓0·68	0·0066	Reduced the growth of a variety of micro‐organisms in studies evaluating secondary metabolites produced by *Pseudomonas fluorescens*	Cheng *et al*. (2015)

a For each metabolite, fold difference is expressed as the scaled relative abundance in LP+RBE over LP. Arrows indicate the direction of change between treatments.

b All metabolites presented have a statistically significant (*P* < 0·05) fold difference as determined by a Welch's two‐sample *t*‐test.

c Indicates compounds that have not been officially confirmed based on a standard, but second‐order identity in Metabolon Inc. library.

### Visualization of metabolic pathways between LP+RBE and LP supernatant treatments

The Cytoscape software visualization tool in MetaboLync © was used to highlight lipids (Fig. 4a) and amino acids/peptides (Fig. 4b) in LP+RBE and LP that contained metabolites with reported antimicrobial functions from Table 4. For a given metabolite, node colour indicated the direction and statistical significance, and node diameter indicated the magnitude of the FD. A pathway enrichment score was calculated for each metabolic pathway as described in the methods. Lipid metabolic pathways visualized in descending order of pathway enrichment score were: hydroxy fatty acids (3·4), long‐chain fatty acids (3·2), dicarboxylate fatty acid (1·7) and inositol (1·1) (Fig. 4a). Amino acid/peptide metabolic pathways visualized, in descending order of pathway enrichment score were: carnitine (2·2), tryptophan (1·7), methionine, cysteine, S‐adenosyl methionine and taurine (1·5), polyamine (0·6), and glycine, serine and threonine (0·4) (Fig. 4b).

## Discussion

This study demonstrated that a synbiotic of *L. paracasei* and RBE contains small molecules with the capacity to reduce *S*. Typhimurium growth, and appeared to function through pH‐independent mechanisms (Figs 1 and 2). The metabolome of LP and LP+RBE identified 84 small molecules that could explain how LP+RBE reduced *S*. Typhimurium growth more effectively than LP (Table 3). The presence of MRS components did not contribute to treatment differences between LP and LP+RBE because each treatment contained identical amounts of MRS media. The MRS and MRS+RBE media had similar compounds identified from both LP and LP+RBE treatments, respectively, and therefore the differential effects of LP and LP+RBE were distinguished by FD in the compounds measured between the two treatment groups. The metabolite differences were measured by the relative abundances for each metabolite, and were attributed to *L. paracasei* metabolism of RBE. Given that these metabolites identified had antimicrobial activities against a variety of pathogens (Table 4), there is promising potential for these supernatants to be used with a broad spectrum of pathogen protection applications relevant to food safety as well as human and animal health.

RBE‐mediated alterations to *L. paracasei* lipid metabolism, primarily fatty acid production, may explain how LP+RBE can reduce *S*. Typhimurium growth more than *L. paracasei* alone. Bacterial production of fatty acids may occur through fermentation of complex carbohydrates, catabolism of complex lipids with fatty acid moieties, de‐novo fatty acid synthesis or via the bioconversion of amino acids into fatty acids (Buccioni *et al*., 2012). Maleate, azelate, alpha‐hydroxyisocaproate, lineolate, oleate/vaccenate and 13‐HODE were abundant in LP+RBE compared to LP and had reported antimicrobial activity. Maleate is commonly used as a bacteriostatic agent against *S*. Typhimurium on frankfurters (Gadang *et al*. 2008). Azelate is used as a topical dermatosis agent to reduce the growth of *Staphylococcus aureus*,* Staphylococcus epidermidis* and *Propionibacterium acnes* (Charnock *et al*. 2004). Both maleate and azelate are present in the rice plant (Kim *et al*., 2014; Agarrwal *et al*., 2016), and *L. paracasei* metabolism increases their bioavailability. Linoleate is also detected at high levels in rice bran (Kaur *et al*., 2012). Multiple species of lactobacilli have the capability to produce linoleic acid, as well as catabolize linoleic acid into oleate/vaccenate and 13‐HODE (Black *et al*. 2013; Druart *et al*. 2014; Pessione, 2012). Collectively, these fatty acids in LP+RBE demonstrated to have bacteriostatic functions against multiple pathogenic micro‐organisms, including *S*. Typhimurium, and this may be mediated in part due to interference with bacterial fatty acid synthesis (Hinton and Ingram 2000; Zheng *et al*. 2005; Martin‐Arjol *et al*. 2010).

In addition to lipids, RBE amino acids and small peptides serve as another metabolite source that *L. paracasei* can bioconvert into different compounds or catabolize into free amino acids with antimicrobial functions (Neis *et al*. 2015). Alterations to methionine and tryptophan metabolism may serve as major mechanisms by which RBE enhances the antimicrobial activity of *L. paracasei* against *S*. Typhimurium. Methionine sulfone (8·29‐fold increase in LP+RBE), which forms during methionine oxidation, has been shown to inhibit *S*. Typhimurium growth by blocking glutamate synthesis (Hentchel and Escalante‐Semerena 2015). Lactobacilli modulate the oxidative state of their environment by producing hydrogen peroxide (Pessione 2012). The supernatant environment created by LP+RBE, coupled with normal hydrogen peroxide production by *L. paracasei* may facilitate methionine oxidation into methionine sulfone. Although methionine sulfone is typically considered a marker of oxidative damage (Hoshi and Heinemann 2001), its presence in controlled amounts may be beneficial as an antimicrobial defence. Multiple studies have investigated the roles of *Lactobacillus* sp. in tryptophan metabolism, which have linked the production of tryptophan‐derived metabolites to modulations of the mucosal immune and enteric nervous systems (Zelante *et al*. 2013; Clarke *et al*. 2014). Indole‐3‐carboxylic acid was increased and is synthesized from tryptophan by both plant and microbial species (Kavitha *et al*. 2010; Böttcher *et al*. 2014). As an antimicrobial agent, indole‐3‐carboxylic acid has been shown to inhibit the growth of a variety of bacterial and fungal pathogens, including multidrug‐resistant bacteria (Zutz *et al*. 2016). In the presence of RBE, *L. paracasei* may increase its production or the bioavailability of indole‐3‐carboxylic acid during tryptophan metabolism.

Metabolism of RBE by *L. paracasei* may have increased the bioavailability of other rice bran phytochemicals, including harmane and 4‐hydroxybenzoate (Table 3). Harmane is a beta‐carboline alkaloid present in plants, and has been shown to have growth inhibitory and bactericidal effects against some *Salmonella* sp. (Arshad *et al*. 2008), and works mechanistically to intercalate DNA (Cowan 1999). 4‐hydroxybenzoate, a derivative of benzoic acid produced by plants and some species of bacteria, has been demonstrated to reduce the growth of a variety of Gram‐positive and ‐negative bacteria, yeasts and moulds (Peng *et al*. 2006; Kosová *et al*. 2015). Increased expression of both hypoxanthine and tricarballylate in LP+RBE suggested that RBE modulations to *L. paracasei* nucleotide and TCA cycle metabolism are important to its *S*. Typhimurium growth‐inhibitory properties (Table 3). Tricarballylate was reported to inhibit citric acid cycle enzymes and was reported to have toxic effects on *S. enterica* when it was accumulated intracellularly at high levels (Boyd *et al*. 2012). Past investigations have associated tricarballylate production with the metabolic activity of healthy intestinal and rumen flora (McDevitt and Goldman 1991; Cook *et al*. 1994), suggesting that in the presence of RBE, *L. paracasei* may increase the production of tricarballylate to levels that *S*. Typhimurium cannot tolerate so that it has a decreased ability to replicate and/or invade host cells.

Four of the lipid and three of the amino acid groups had pathway enrichment scores greater than one, indicating these pathways contribute to LP+RBE effects on growth inhibition. For example, spermine and pinitol decreased 0·70‐fold and 0·61‐fold in LP+RBE, yet in past investigations they increased the function of methicillin and beta‐lactam antimicrobials respectively (Kwon and Lu 2007; Lievin‐Le Moal *et al*., 2011; Ahmad *et al*. 2016). These studies suggest that regardless of absolute or relative metabolite abundance, the mere presence or absence of metabolites within the supernatant influence the functions of other metabolites present in the supernatant. The level of inhibition, antagonism, additive or synergistic functions for a given combination of metabolites in the supernatant is complex to investigate as a bioactive mixture, and the reductionist approaches needed may yield conflicting results. Thus, the relationships between metabolites, for inhibition of pathogen growth, remains an area of ongoing investigation that can be further explored with targeted metabolite profile analyses and merits consideration of bioactivity‐guided fractionation methods. The metabolomics investigation conducted herein compared the mechanisms of LP+RBE and LP alone by looking at levels of metabolite abundance and metabolic pathway activity. In order to distinguish the unique mechanisms by which RBE modulates *L. paracasei* metabolism, the metabolome could be compared with gene expression levels obtained from metatranscriptomics and genomics analyses of LP+RBE and LP. This approach would seek to determine direct changes to *L. paracasei* gene expression of metabolic enzymes in the presence of RBE. Evaluating the small‐molecule profiles of a symbiotic should integrate information from metabolites, overlapping pathways and metabolic networks to provide a comprehensive understanding of overall function.

The experiments described herein illustrated the *in vitro* efficacy of LP+RBE over LP alone in reducing *S*. Typhimurium growth, and warrant investigation for the efficacy of LP+RBE against other enteric pathogens. These data, demonstrating enhanced antimicrobial activity of LP+RBE, support results from various animal models (Kumar *et al*. 2012; Yang *et al*. 2015). In mice, rice bran simultaneously reduced *S*. Typhimurium shedding and promoted *Lactobacillus* growth (Kumar *et al*. 2012), and in pigs, there was less diarrhoea from Rotavirus (Yang *et al*. 2015), suggesting that LP+RBE may benefit the host for prevention of pathogen colonization, and for the host immune system to mount a protective response before the onset of extensive disease. The dose of RBE used in these studies has influenced the observable levels of pathogen inhibition, and future investigations merit attention to RBE dose with an intact and varied microbiome composition and structure for demonstrating antimicrobial activities with *L. paracasei*. Given the growing concern for antimicrobial resistance in *S*. Typhimurium, alternative treatments and preventive measures are needed. Understanding the metabolite profile of a synbiotic can lead to a comprehensive understanding for how food components enhance the natural antimicrobial activity of *L. paracasei*. In an era of multidrug resistance, synbiotics of *Lactobacillus* and rice bran have strong potential to provide broad‐spectrum protection against many pathogens by serving as viable, natural alternatives or by applications in combination with standard antimicrobial drugs to reduce the dosages needed to be efficacious.

## Conflict of Interest

No conflict of interest declared.

## Supporting information


**Figure S1** Dose–response studies were performed for selection of (a) RBE concentration and (b) vehicle control volume to use in the *S*. Typhimurium growth reduction assay.
