# Supplementary figures and images for: Lactobacillus paracasei metabolism of rice bran reveals metabolome associated with Salmonella Typhimurium growth reduction
Source: J Appl Microbiol. 2017 May 11;122(6):1639–56. doi: 10.1111/jam.13459 (PMC5518229; doi:10.1111/jam.13459)

Supplementary Figure 1.

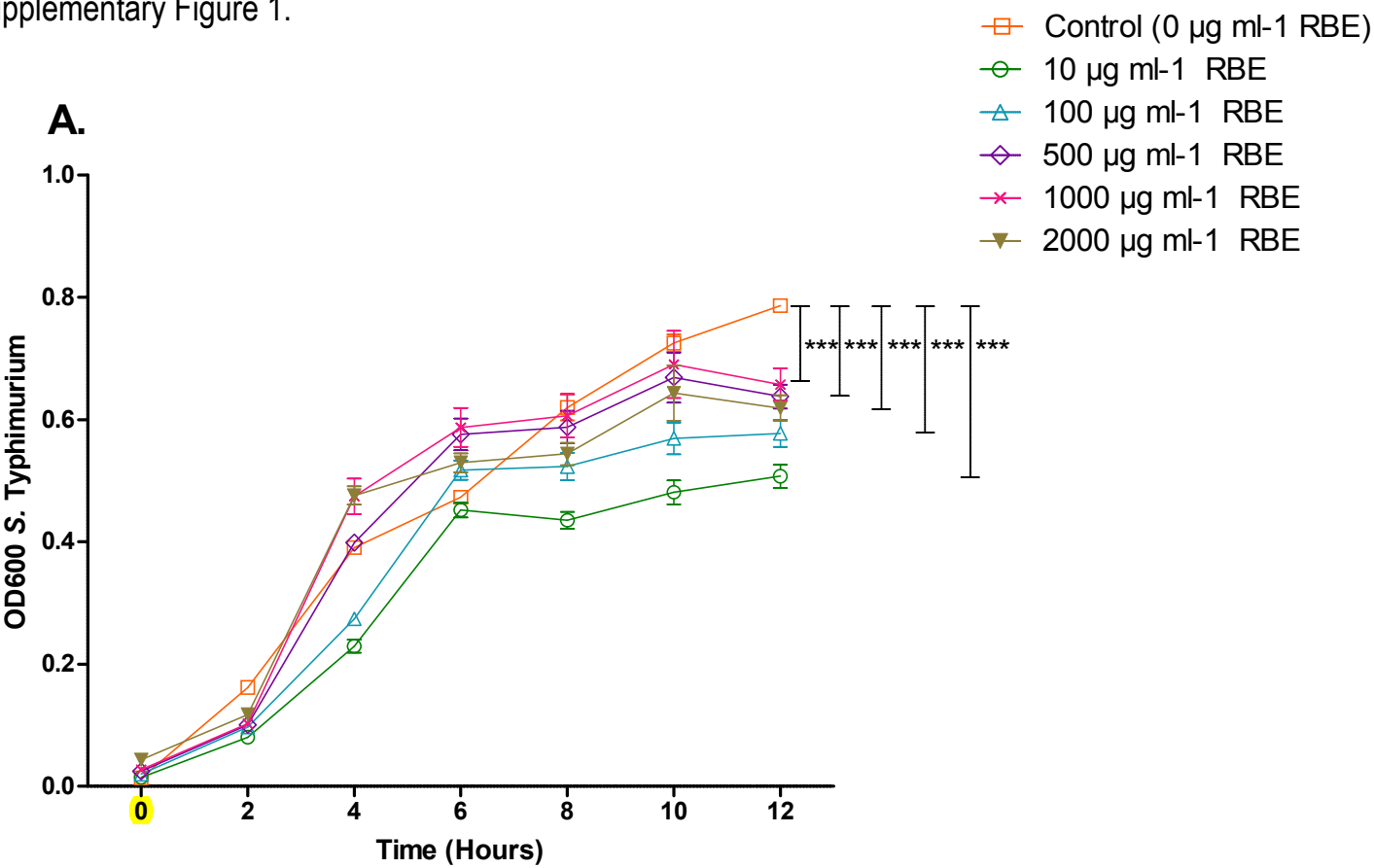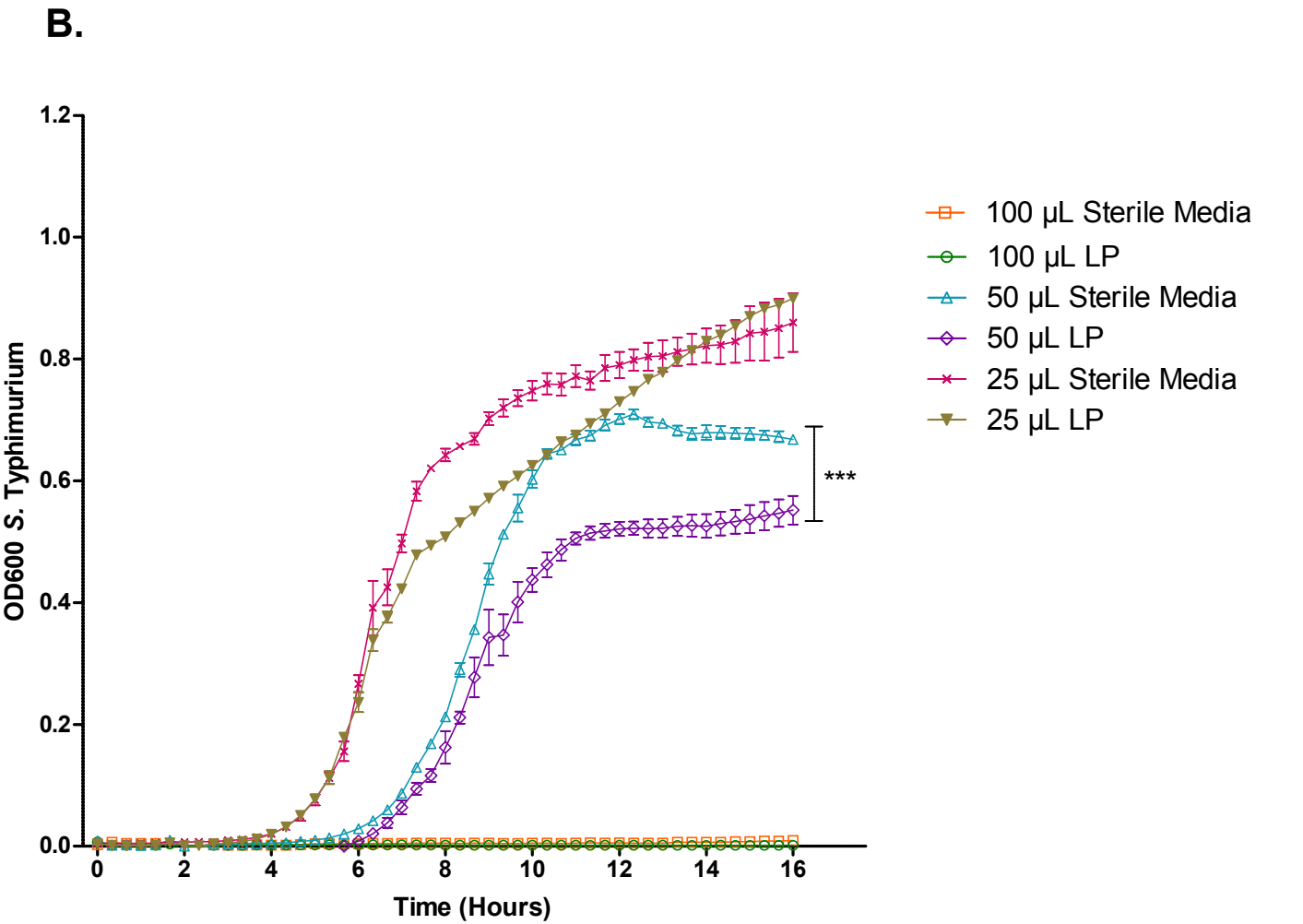

Supplement: Supplementary file 1 — Figure S1 Dose–response studies were performed for selection of (a) RBE concentration and (b) vehicle control volume to use in the S. Typhimurium growth reduction assay. [file JAM-122-1639-s001.pdf]
